# Why isn’t sex optional? Stem-cell competition, loss of regenerative capacity, and cancer in metazoan evolution

**DOI:** 10.1080/19420889.2020.1838809

**Published:** 2020-12-10

**Authors:** Chris Fields, Michael Levin

**Affiliations:** a Caunes Minervois, France; b Allen Discovery Center at Tufts University, Medford, MA, USA

**Keywords:** Evo-devo, facultative sexuality, germline progenitors, Hamilton’s rule, PIWI/piRNA system, whole-body regeneration

## Abstract

Animals that can reproduce vegetatively by fission or budding and also sexually via specialized gametes are found in all five primary animal lineages (Bilateria, Cnidaria, Ctenophora, Placozoa, Porifera). Many bilaterian lineages, including roundworms, insects, and most chordates, have lost the capability of vegetative reproduction and are obligately gametic. We suggest a developmental explanation for this evolutionary phenomenon: obligate gametic reproduction is the result of germline stem cells winning a winner-take-all competition with non-germline stem cells for control of reproduction and hence lineage survival. We develop this suggestion by extending Hamilton’s rule, which factors the relatedness between parties into the cost/benefit analysis that underpins cooperative behaviors, to include similarity of cellular state. We show how coercive or deceptive cell-cell signaling can be used to make costly cooperative behaviors appear less costly to the cooperating party. We then show how competition between stem-cell lineages can render an ancestral combination of vegetative reproduction with facultative sex unstable, with one or the other process driven to extinction. The increased susceptibility to cancer observed in obligately-sexual lineages is, we suggest, a side-effect of deceptive signaling that is exacerbated by the loss of whole-body regenerative abilities. We suggest a variety of experimental approaches for testing our predictions.

## Introduction

For many animals, sex is optional. Placozoa [1], sponges [2], ctenophores [3], cnidarians [4], basal bilaterians including acoels [5], various invertebrates including flatworms [6] and annelids [7], and even basal chordates, the colonial ascidians [8] provide examples of vegetative (i.e. agametic) reproduction by budding, fission, or fragmentation accompanied by whole-body regeneration (WBR) in species that are also sexually competent [see 9–11 for comparative analysis]. Many other invertebrates, including some insects [11] and snakes [12], are capable of hermaphroditic self-fertilization or parthenogenesis, often facultatively. While a number of theories, including the Fisher–Muller hypothesis [13], Muller’s ratchet [14], and Red Queen dynamics, in which competitive “arms races” continue indefinitely [15], have been proposed to explain the prevalence of sex despite its costs, recent models suggest that facultative sex combined with either vegetative reproduction, self-fertilization, or parthenogenesis can achieve the benefits of obligate sex for genetic diversity with greatly reduced costs, making it potentially an optimal reproductive strategy [16–18]. Facultative sex is not, however, the observed strategy for most anatomically complex animals, including mammals. Why not?

The question of why any animals would be obligately sexual can be asked in at least two distinct ways. Acknowledging that many extant lineages are in fact obligately sexual, one can ask how, given its costs, this condition is maintained. In lineages with long histories of obligate sexuality such as mammals, developmental, physiological, or behavioral constraints may prevent reversion to asexual reproduction with facultative sex, so this question is of greatest interest in lineages with close facultatively sexual relatives. The standard answer to this question is that the costs of sex to the individual organism, including the costs of building and maintaining specialized sexual structures and a germ line, acquiring the genetic and epigenetic capability to build a complete organism from one cell, and engaging in the social interactions required for mating [see also [19], for a discussion of more subtle costs], are paid for by the decrease in individual-level genetic diversity and increase in population-level genetic diversity that sex enables. Intense pressure from rapidly-evolving parasites leading to Red Queen dynamics [20–22] and sexual conflict suppressing the fitness of asexual variants [23] are recent versions of this answer [see [24], for evidence that the latter mechanism can also drive populations toward asexuality].

A different question, one that applies equally to all obligately sexual lineages, is how obligate sexuality could arise in the first place. No known unicellular organisms are obligately sexual; indeed obligate sexuality is rare among animals outside of the bilaterians. In the context of basal metazoan evolution, this question can be posed particularly sharply: what selective pressure(s) could first drive vegetative reproducers that suffer none of the specific costs of sex to extinction, replacing them with obligate gametic reproducers, and then drive hermaphroditic self-fertilizers and parthenogenic reproducers to extinction, replacing them with obligate sexuals? Thomas *et al.* [25] have recently suggested that transmissible cancers may exert sufficiently strong selective pressure against asexuality in all forms, including self-fertilization and parthenogenesis, with obligate sex providing the only means of generating sufficient genetic diversity, and hence a sufficiently different “self” in each generation, to allow an effective immune response. As discussed below, however, obligate sex positively correlates, across animal lineages, with susceptibility to cancers [26, 27].

As Lai and Aboobaker [9] point out, WBR strongly correlates with the presence of non-germline stem cells expressing components of the hypothesized germline multipotency program [GMP; 28], including the PIWI/piRNA transposon repression system [29,30], *vasa* [31], *nanos* [32], *tudor* [33], and other typically germline regulators. At least in flatworms [34] and annelids [7], vegetative reproduction also requires specific behaviors (e.g. to induce fission) that can be lost separately. As non-germline stem cell populations are required for tissue homeostasis in multicellular organisms [35], the specific cost of asexual reproduction via WBR is the cost of these reproductive behaviors, a cost that is avoided if WBR follows injury. Setting behavioral considerations aside and focusing on WBR only, the question of how obligate gametic reproduction arose in the first place can be framed in molecular terms: what selection pressure(s) could sufficiently repress the GMP in non-germline stem cells to render WBR no longer possible? What selection pressure(s), in other words, led to the loss of WBR in lineages that were thereby rendered obligately gametic? This way of formulating the question is consistent with the idea that multi- or totipotent stem cells are ancestral, and give rise in some lineages to germline-specific stem cells that may (in facultative sexuals) or may not (in obligate sexuals) co-occur with non-germline stem cells [36]. It suggests that stemness is a default state that must be actively repressed outside the germline if gametic reproduction is to be obligatory. How does this repression happen?

If individual organisms are assumed to be maximal units of cellular cooperation [37] and cooperation is assumed to be proportional to genetic relatedness [[38], we discuss below reasons to reject both of these assumptions], obligate sexuality emerges in models that assume early sequestration and a low mutation rate in germline stem cells [39]. Obligate sexuality is, in such models, a conflict-resolution mechanism; it prevents “defectors” – somatic cells that may acquire mutations that decrease cooperativity, as in cancers – from reproductively competing with the organism as a whole [39,40]. From the perspective of stem-cell lineages, however, the fitness of a sexual individual is the fitness of its gametes, and the fitness of an asexual individual is the fitness of its WBR-capable stem cell population. A gamete is moreover, from this perspective, a stem cell that has “defected” from its responsibility, as part of the cooperative organism-scale individual, for maintaining tissue-level homeostasis and instead isolated itself within a protective microenvironment, the gonad, that has the sole function of preserving its reproductive fitness. Obligate sexuality emerges, on this view, in any lineage in which such defection is advantageous to the defector.

**Figure 1.**
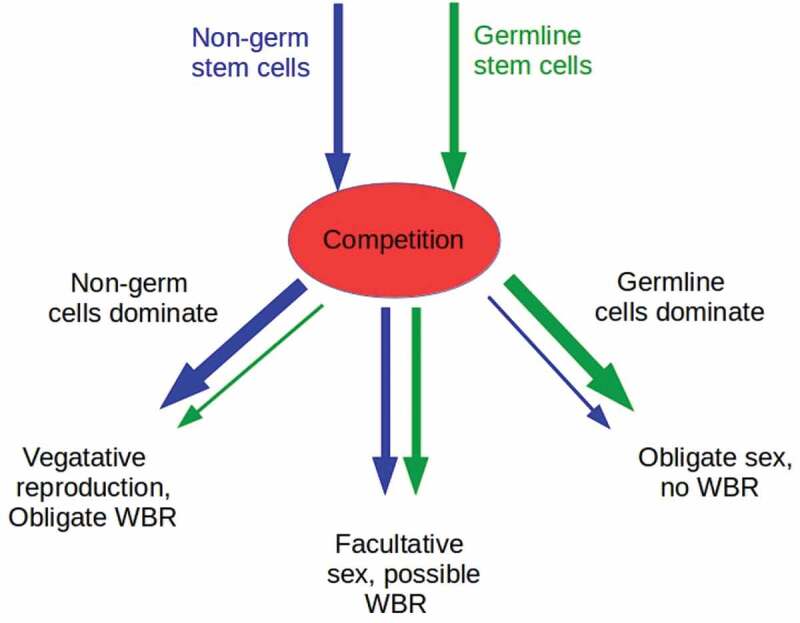
Competition between non-germ and germline stem cells can lead to three classes of outcomes. If non-germ cells dominate and suppress the proliferation of germ cells, animals can be expected to be vegetative reproducers with obligate WBR to replace missing structures following fission or damage (left branch of diagram). If germline cells dominate and suppress the proliferation or totipotency of non-germ cells, animals can be expected to be obligate gametic reproducers with at least one sex, the female, if parthenogenic (right branch of diagram). If neither dominates, or if dominance is only partial, animals can be expected to be facultatively sexual, and capable of vegetative reproduction with WBR, parthenogenesis, or both. Similar competitive mechanisms may operate in plants, though we do not consider these here

In line with this view of germline stem cells as defectors, we here suggest that obligate gametic reproduction (hereafter “sexuality” except where hermaphroditic self-fertilization or parthenogenesis must be distinguished for clarity) arose in animals not as a response to any external threat, but as a result of runaway competition between distinct stem cell lineages. Specifically, we consider competition between totipotent (i.e. GMP-competent) germline and non-germline stem-cell lineages in the context of an “imperial” model of multicellularity [41,42] in which the multicellular state is stable only if the proliferative capacity of non-stem lineages is actively suppressed. If germline and non-germline stem cells do not compete or compete only minimally, facultatively sexual systems also capable of vegetative reproduction and WBR from fragments, as observed throughout the basal metazoa, can be expected (Figure 1). Inter-lineage competition for resources, and for control of resource-delivering somatic cells can, however, be expected, and natural selection would, as it does at the organism level, amplify any genetic or epigenetic differences that enabled such competition. If non-germline stem cells “win” and suppress germline development, obligate vegetative asexuals that altogether lack specialized germline stem cells, gametes, or gonads, such as the laboratory model planaria *Dugesia japonica* or *Schmidtea mediterranea* can be expected. Population variants or close relatives of both of these species are sexual, suggesting that full suppression of germline development is not stable over evolutionary timescales [34,43]. If, on the other hand, germline stem cells “win” and suppress totipotency in non-germline stem cells, obligate gametic reproducers incapable of WBR can be expected. Obligate sexuality in the strict sense would then result from any mechanism, e.g. incompatibility between male and female gonadal structures or competition between inducers of meiotic versus mitotic division, that suppressed self-fertilization or parthenogenesis. Germline – non-germline competition has been proposed before, in the context of the “disposable soma” model of organismal senescence [19,44], but as a result of obligate sexuality, not as a cause. Here we suggest the reverse.

The strongly negative correlation, observed throughout animal phylogeny, between regenerative ability, particularly WBR, and obligate sexuality results immediately in this model: gametic reproduction is the only option available once WBR has been lost. This competition-based model also offers an explanation for the observed correlation between obligate sexuality and both the frequency and severity of cancers. While cancers have been observed in cnidarians and morphologically less-complex bilaterians including flatworms, they are much more common in anatomically more-complex animals, the vast majority of which are obligately sexual [26,27]. Both modeling studies and endocrinology suggest that selection for fertility can increase cancer susceptibility [45,46]. Dysregulation of the PIWI/piRNA system also correlates with cancer [47]. On the other hand, regeneration, especially WBR, can clear or reprogram cancer cells [reviewed in [48, 49]]. As described in detail below, germ cells can directly induce cancer in the current model by dysregulating non-germ stem cells. Hence a correlation between obligate germline reproduction, even if parthenogenic, and cancer is to be expected.

We focus here on the transition between vegetative reproduction with facultative sex as observed throughout the basal metazoa, and develop a competition-based model in three stages. The first is to reexamine development from the perspective of a single stem-cell lineage. In the imperial model, the task of a stem cell lineage is to produce a microenvironment conducive to its own indefinite reproductive survival, i.e. to its own fitness [41,42]. To do this, it employs all of the information that it can acquire, whether from its genome or other cellular memory structures or from its environment [50]. Stem-cell lineages can, therefore, compete for dominance by attempting to control access to information as well as access to energetic resources. The second step is to interpret the “relatedness” *r* in Hamilton’s rule [38] and its extensions [51] broadly to include informational similarity at all scales, not just genetic relatedness. The existence of chimeric organisms, multi-species microbial mat communities, symbiotic associations, and obligate eukaryote-prokaryote holobionts [52,53] all indicate that cooperation depends on more than simply genetic relatedness. Lineages dependent on the long-term maintenance of surrounding somatic tissues (i.e. the body) share, on this extended measure, more relatedness that lineages that are not so dependent, i.e. germ-cell lineages for which the future lies outside the body. Lineages promoting meiosis are, similarly, outliers among lineages promoting mitosis. The cooperation between soma-dependent and germline lineages required to support sexual reproduction can, we suggest, only be obtained by the use of coercive and deceptive signaling that hides its true cost to the cooperating somatic cells. The third step is to show that, with reasonable assumptions, winner-take-all competition between germline and non-germline stem lineages arises and renders facultatively sexual strategies unstable equilibria that can be driven toward obligate sexuality or asexuality by minor perturbations. Evidence that Wnt pathway activity can act as a switch between WBR capability and obligate sex in planaria [reviewed by 43], discussed further below, supports this step. We then consider PIWI/piRNA system dysregulation in non-germline lineages as a plausible competitive tactic on the part of germline stem cells. Cancer is, in this case, an unintended but effective means for “disposing” of the soma; regeneration may counteract this process by over-riding dysregulatory signals. We close with suggestions for experimental tests.

## Building a body is building an environment

Development is most commonly thought about from a whole-organism perspective: when, where, and how individual cells divide, migrate, differentiate, or die is generally analyzed within the context of an organism-level developmental process that is either “normal” or aberrant in some way. From the perspective of a single cell, however, development is a succession of changes in its microenvironment. Setting aside migration, a proliferative cell constructs its microenvironment out of its non-proliferative progeny [41,42]. The metazoan body is, in this case, the combined environment inhabited and maintained by its proliferative cells, in particular its stem cells.

Considering a single, proliferative stem cell and assuming for simplicity that active migration is negligible, the “choices” available at any given moment are whether to divide and what, if any, signals to send to nearby cells. These choices are made on the basis of internal and locally-available external information, including genomic information via transcriptional and hence proteomic state, available energy via metabolic state, and the activity and differentiation status of nearby cells via the molecular, bioelectric, contact, or other signals that they provide. The transition from “development” to “maintenance” for a single proliferative cell occurs when the local microenvironment achieves a mostly-steady state. This transition may or may not correlate with the organism-scale “target morphology” – the morphology at which organism-scale development or regeneration stops in “normal” individuals [54] – being reached; unless stem-cell lineages are somehow globally synchronized, lineages in different parts of the body can be expected to reach the maintenance stage at different times.

Organism-scale bodies hold together as coherent, bounded individuals because cellular-scale microenvironments overlap; disruption of this overlapping of microenvironments results in organismal dissociation, e.g. in identical twins when an early mammalian embryo partially dissociates. Microenvironmental overlap enables communication between proliferative stem cells, which may be from the same or from different proximate lineages and which may, therefore, have more or less similar cellular states. Friston *et al*. [55] analyzed models in which “cells” both communicate their current states to their neighbors and update their states based on the signals received from their neighbors, finding that they are able to self-organize into a “body” with a specific target morphology [see also 56,57,58]. Modifying the interpretation of the signals received, in this model, leads to dysmorphologies [55,59]. The “cells” in these models are Bayesian agents employing active inference [60,61]; they have both long-term (genetic) and short-term (cellular state) memories, inferential and communication capabilities, and the ability to modify their local microenvironments by moving in space relative to the other cells. They therefore exhibit a kind of “basal cognition” [62,63] in the sense of systems that exhibit memories and make decisions about possible outcomes in a context-specific manner. In these models, communication is cooperative: the cells do not withhold information, provide misinformation, manipulate each other’s information processing, or send signals telling other cells to stop dividing or die. We examine the consequences of relaxing these restrictions on communication below.

In animals as well as plants, germline cells inhabit specialized gonadal microenvironments constructed out of somatic cells. In *C. elegans*, for example, the gonadal primordium at hatching comprises four cells: two germline stem cells and two somatic progenitor cells derived from the MS lineage [64]. The two somatic progenitor cells undergo sex-specific, (mostly) invariant sequences of divisions to produce progeny that differentiate to form a male gonad in males and both male and female gonads in hermaphrodites [65]. In *Drosophila*, both germline stem cells and somatic gonadal precursors are born at dispersed locations in the early embryo and aggregate to form the embryonic gonad, which differentiates to either male or female during postembryonic development [66]. In vertebrates, somatic gonadal precursors aggregate first to form a bipotential embryonic gonad, to which germline stem cells migrate prior to sex determination and differentiation into male- or female-specific structures and germ cells [67], see [68, 69], for comparative reviews of vertebrate and invertebrate strategies]. In all of these systems, the somatic gonad develops even in the absence of germline cells. Hence an obligate sexual system involves not just the differentiation of specific germline stem cells, but also specific somatic gonadal precursors. Isolation of germline cells within specialized gonadal tissues, including physical separation between male and female gonads in hermaphrodites, limits their communication with non-gonadal lineages, and with each other, to long-range signals such as circulating hormones.

The genetic interest of any stem cell lineage is effective immortality within its microenvironment. While non-germline stem cells construct and maintain their own microenvironments, germline stem cells are dependent on non-germline cells to construct and maintain their microenvironments. This asymmetry in control creates an asymmetry of interests: germline stem cells have a vital interest in controlling the behavior of the non-germline cells on which the integrities of their microenvironments depend. This asymmetry of interests sets the stage for competition. We first consider this asymmetry-driven competition in general, then focus on potential mechanisms for direct, winner-take-all competition between totipotent stem lineages for control of reproduction in an ancestral metazoan capable of both vegetative and sexual reproduction.

## Germ and non-germ lineages compete for “spheres of influence”

While individual animal bodies have been proposed to be loci of maximal cooperation [37], cells and cell lineages within individual bodies nonetheless compete for energetic resources, trophic factors, and space, with cells perceived by their neighbors to be less fit being actively eliminated [70–73]. Intercellular competition between germline cells and their associated gonadal tissues, on the one hand, and non-gonadal somatic tissues on the other leads, in “Y-models” of resource allocation, to post-reproductive somatic senescence [19]. In *C. elegans*, germ cells are directly implicated in somatic senescence via systemic metabolic regulation; ablating the germ cells extends somatic lifespan by up to a factor of two [74,75].

Observations in planaria suggest a more subtle form of competition between germline and non-germline stem cells. Asexual planaria can be sexualized by feeding them sexual planaria [76,77] or by transplanting totipotent stem cells (neoblasts) from sexual planaria into them [77,78]. Unlike neoblasts of asexual planaria, those of sexual planaria both encode and express instructions for making germ cells and gonadal structures. Sexualization (in this case, to cross-fertilizing simultaneous hermaphrodites) forces reproduction through a zygotic bottleneck, and hence exclusively favors the progeny of germline lineages. While the sexualization mechanism in this case remains unknown, up-regulation of canonical Wnt pathway activity has been shown to both suppress regeneration and sexualize in other planarian species [79,80]. On the basis of these results, Vila-Farré and Rink [43] have suggested that Wnt pathway activity, which is known to be essential for the regeneration of posterior structures in planaria, may also serve as a switch between gametic and vegetative reproduction. As Wnt is a versatile, early- as well as late-acting regulator of both polarity and cell fate across multicellular animals [108], confirmation of a mechanistic link between Wnt activity, regeneration, and sexualization outside of the planaria would provide broader evidence for the competitive model proposed here.

Cell-cell competition via morphogens such as Wnt, or in mammalian cells Myc [81], is effectively competition to determine cell fate. It is perhaps useful to think of such competition as *political*: it extends beyond the control of access to and allocation of resources (i.e. “economic” competition) described by Y models into the domain of access to information and even control of how available information is interpreted [82]. It raises the possibility of cells using what amounts to disinformation to modulate the differentiation status of neighboring cells to their own advantage. Deception is well-characterized at the organismal level and extends throughout animal and even plant phylogeny [83]; we suggest here that it is also employed at the cellular scale, for the same reasons of selective advantage against competitors.

To make this suggestion more precise, it is useful to start with Hamilton's rule [38]: that natural selection will favor cooperative behaviors only if *rb – c* > 0, where *r* measures the relatedness between parties, *b* is the fitness benefit to the recipient of cooperation, and *c* is the cost to the cooperator. The “relatedness” *r* is standardly taken to be the genetic relatedness; hence family members are more likely to cooperate than strangers [see [51], for extensions that model within-family conflict]. The standard meaning of “fitness” is the probability of gene transfer to the next generation, i.e. a sexual reproduction system in which individual-organism death is assumed. If the two parties are clones, *r* = 1 and the rule is just a cost-benefit tradeoff. Cooperation between sister cells, neither of which can survive outside a multicellular body, clearly satisfies *b – c* > 0 if the recipient of cooperation is a germ cell and the cooperator is a somatic cell that helps assure germ-cell survival. It is satisfied even if the cost *c* is cell death: the somatic cell will die anyway, and germ-cell survival is its only chance of a genetic contribution to the next generation. The possibility of somatic-cell survival eliminates this benefit, and with it any motivation for somatic-cell cooperation; hence non-germline stem cells are not expected, on the basis of Hamilton’s rule alone, to cooperate with the germline in systems with robust WBR.

**Figure 2.**
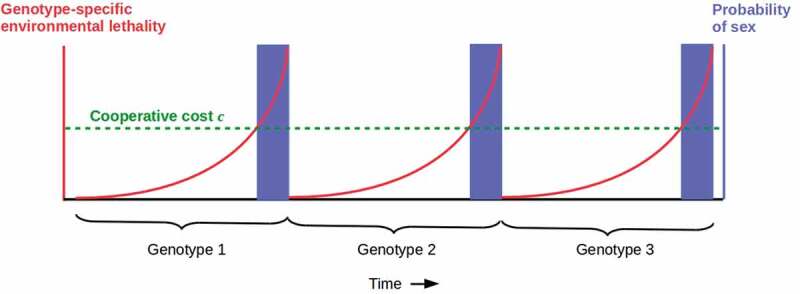
Simplified model of alternating asexual and sexual reproduction driven by genotype-specific environmental lethality due e.g. to parasitism. Assuming genotype-specific lethality is sufficient to completely eliminate the susceptible genotype, species survival requires sex to generate a (one, for simplicity) new genotype. In this case, the Hamilton’s rule benefit *b* for somatic cells of cooperating with germ cells to enable sex rises with lethality; in the simplest case, they are the same (red) curve. When the cost threshold *c* sufficient to enable sex (green dashed line) is reached, sexual reproduction occurs (blue bars) until the existing genotype is eliminated by the still-rising environmental lethality. The cycle then repeats with the new, sexually-generated genotype

Fitness considerations beyond Hamilton’s rule, e.g. of Red Queen dynamics driven by external threats, may still induce somatic cells to cooperate with germ cells to produce genetically less-related offspring even in the presence of vegetative reproduction with WBR. Suppose that for any given genotype, the lethality of the environment increases monotonically but slowly compared to the generation time (Figure 2). Suppose further that germ cells cannot contribute DNA to the next generation, i.e. sex is disabled, below some fixed total cost *c* contributed collectively by some number of somatic cells, but that sex occurs with probability one whenever *c* is exceeded. In this case, the benefit *b* of cooperation is time-dependent, *b*(*t*) and tracks the increase in environmental lethality. A phase transition to cooperation occurs when *b*(*t*) > *c*. If many somatic cells contribute to meeting the cost *c*, the phase transition can be described in terms of percolation theory [40]. Cooperation enables sex and hence a new genotype for which lethality is lower, and the cycle repeats itself. Such cyclic sexuality occurs in planaria [32,41], though single populations that alternate between sexual and asexual (vegetative) reproduction appear to be rare.

Let us now consider cooperation not by somatic cells to benefit germ cells, but by germ cells to benefit somatic cells, again assuming *r* = 1. In a vegetative system, germ calls may not be present; this is the case in fissiparous planaria. If they are present, and are regenerated as needed following fission, fragmentation, or budding, their genes are transmitted to the next generation by their somatic sisters. The case of interest is the sexual one: here germ cells have a fitness motivation to cooperate with somatic cells only to assure that they receives the resources for, and only up to the point of, successful sexual reproduction. Germ cells in sexual systems have nothing to lose from a post-reproductive disposable soma, with a semelparous lifecycle as the extreme case [44]. Hermaphroditic *C. elegans* strains with vulval dysfunctions that prevent egg laying provide a striking example: the unlaid eggs hatch internally, with the next generation emerging after devouring the “disposable” mother [84].

## Cells can enforce cooperation via coercive and deceptive signaling

Reproduction is costly; hence reproductive cells have a fitness motivation to assure adequate resource contributions from somatic cells at minimal cost to themselves. As in organism-scale social relations, deception and coercion are obvious solutions to this cost-minimization problem. Intercellular signals that influence proliferation and differentiation provide an inexpensive medium for both. Signaling with ligands for dependence receptors provides a particularly effective solution, as withholding the ligand “punishes” non-compliant cells by inducing apoptosis [85,86].

The evolutionary motivations for coercive and deceptive signaling become clearer when the concept of relatedness in Hamilton’s rule is extended from the standard, strictly genetic measure to a similarity measure based on cellular state. Despite their genetic relatedness, germline and somatic cells, including lineage-committed non-germline stem cells, are in fundamentally different states [87]. Transcriptome, proteome, and “architectome” – the state of the cytoplasm, cytoskeleton, and membrane – encode far more information than the genome [50]. Whole-organism scRNA sequencing studies show that germline cells are transcriptional outliers in *C. elegans* [88], *Drosophila* [89], zebrafish [90] and *Xenopus* [91]. Pluripotent stem cells are similarly transcriptionally distinct from differentiated cells in sponges [92], the cnidarian *Nemostella* [93], and planaria [94]. Pluripotent human embryonic stem cells express a densely-connected network of cytoplasmic and nuclear regulatory proteins, again in a distinct pattern from non-pluripotent cells [95]. As noted earlier, the GMP system, including its wealth of small regulatory RNAs, accounts for some of this difference. In part due to the activity of this system, germline stem/progenitor cells are in a state of overall relative transcriptional repression that prevents expression of soma-specific genes [68]. Hence even just taking transcription into account, the cellular-state relatedness *r_cell_* ≪ 1 when comparing totipotent or pluripotent cells to lineage-committed non-germline stem cells or differentiated somatic cells.

If we consider an extended Hamilton’s rule, *r_cell_ b – c* > 0 to be criterial for cooperation between somatic and totipotent stem cells, the cost *c* for the cooperating somatic cell must be *c* ≪ *b* for cooperation to occur. The cost of supporting a germline and its supporting gonads is, however, not trivial even at the level of a single cell; most body mass and hence energy consumption is devoted to gonadal tissue and eggs in some animals, e.g. *C. elegans* hermaphrodites, and gametic sex always correlates with eventual somatic-cell death. “Convincing” somatic cells to bear this cost requires making *c* “look small” to the cooperating cell even when it is in fact large. Conversely, the true cost of sending the coercive or deceptive signal for the germ cell seeking resources must also be small.

When germline stem cells are recognized as defectors from the common stem-cell responsibility of tissue homeostasis, the differential motivation for germline stem cells to employ deceptive signaling becomes clear. Non-germline stem cells, even if totipotent, must cooperate with each other to maintain a body of the right size and shape. Accurate signaling between stem cells is essential for this task. Germline stem cells, as self-isolated defectors from the task of tissue homeostasis, have no need for accurate signaling with non-germline stem cells. The can, therefore, send signals that dysregulate tissue homeostasis, and even lead to organismal death, provided only that the organism continue functioning well enough to reproduce. The rapid degeneration of the adult body following reproduction observed in semelparity provides evidence for such reproductively-coupled dysregulation. Teratomas can, in this model, be interpreted as outcomes of “internecine” competitive dysregulation within the germline stem cell population itself.

As in social interactions, one way to decrease the cost of sending a signal is to employ intermediaries. In the case of germ cells signaling for germline-supporting resources, the most readily available intermediaries are the somatic cells of the gonad. Hence one could expect, on cost-benefit grounds alone, that germline cells would employ inexpensive local signals to direct somatic gonadal cells to implement the energetically more expensive task of manufacturing and transmitting systemic regulatory signals – e.g. sex hormones – to the entire rest of the organism. The relative benefit of doing so, for the germline, would be expected to increase as the size and cell-type diversity of the somatic “host” increases.

## Winner-take-all competition renders facultative strategies unstable

Red Queen dynamics driven by external threats can render vegetative reproduction unstable, as discussed in connection with Figure 2 above. Here we suggest that internal, inter-lineage arms races between stem cells can render facultative sexual strategies unstable. Once established as an independent lineage, we suggest, germ cells will become implacable competitors for somatic stem cells [41]. The results of this competition include loss of WBR capability and increased susceptibility to cancer. We focus on the former in this section, and the latter in the next.

Vegetative reproduction with occasional sexual reproduction appears to be stable in non-bilaterian animals and in basal bilaterians including acoels as noted above [9,10]. Vegetative reproduction and WBR are rapidly lost with increasing morphological complexity, with only isolated hold-outs such as the asexual planaria, fissiparous annelids, or colonial ascidians maintaining these capabilities. What happened, one or more times in the bilaterian lineage, early in or even before the Cambrian Explosion, that rendered most bilaterians obligately gametic reproducers? While developmental regulators such as Hox genes diversify with increasing morphological complexity [96], such diversification does not by itself explain the loss of vegetative reproduction and WBR. Obligate sexual species exist among the planaria, for example, with no evidence for greater diversification of developmental “toolkit” genes. As mentioned above, merely up-regulating β-catenin expression in the posterior half of the animal appears sufficient to both induce gonad development and disable WBR from posterior fragments [79,80].

**Figure 3.**
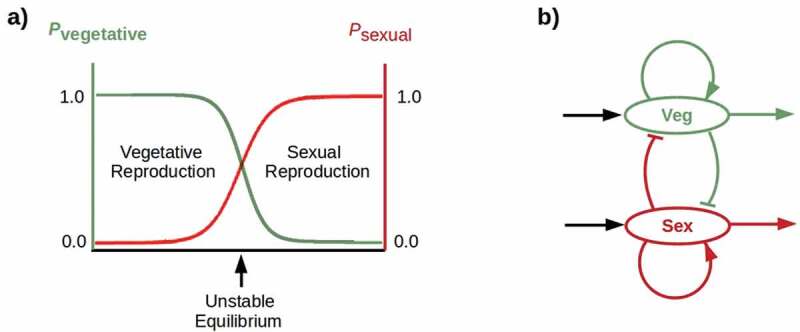
A) Winner-take-all competition: probabilities (*p*) of vegetative (green) and sexual (red) reproduction cross at an unstable equilibrium. b) A simple “flip-flop” circuit implements winner-take-all competition

If germline cells engage in winner-take-all competition for control of reproduction with the non-germline stem cells required for WBR, the facultative strategy observed in non-bilaterian animals becomes unstable (Figure 3). Hence a winner-take-all mechanism may be the innovation that assures obligate sexuality in morphologically-complex bilaterians. Regulated expression of β-catenin provides such a mechanism in planaria: relatively low posterior [β-catenin] enables head regeneration from posterior fragments but disables gonad development, while relatively high posterior [β-catenin] does the reverse. Once established, obligate sexuality can be “locked in” by mechanisms that prevent regression to an asexual state. “Addicting” somatic cells to gonadally-generated ligands, such as androgens [83], that bind dependence receptors renders regression lethal to the addicted cells. Sexualization of the brain resulting in sexual competition or behaviors enabling sexual selection would also “lock in” sexuality. Hence while loss of WBR capability may be a fitness-decreasing event, and some lineages that lose WBR capability may thereby suffer extinction, lineages that survive the loss may be incapable of regaining WBR, and may in consequence be subjected to extreme selection for sexual efficiency. Organisms like *C. elegans* that lack non-germline stem cells, are incapable tissue replacement, and devote all available resources to reproduction may be indicative of such a history of selection.

What could trigger winner-take-all competition? External threats leading to Red Queen dynamics may select for frequent sex, but do not rule out facultative sex [16–18]. External threats do not, moreover, explain the loss of WBR capability. As noted earlier, any imbalance in access to resources between stem-cell lineages could trigger competition, with defection of the presumptive germline lineage to a protective, resource-supplying gonadal environment as a possible outcome. However, for such defection to be successful, i.e. for gametic reproduction to become obligatory, a mechanism that suppresses totipotency outside the germline is needed. Such a mechanism would then be expected, under continuing selection, to be hard-coded into embryonic development.

**Figure 4.**
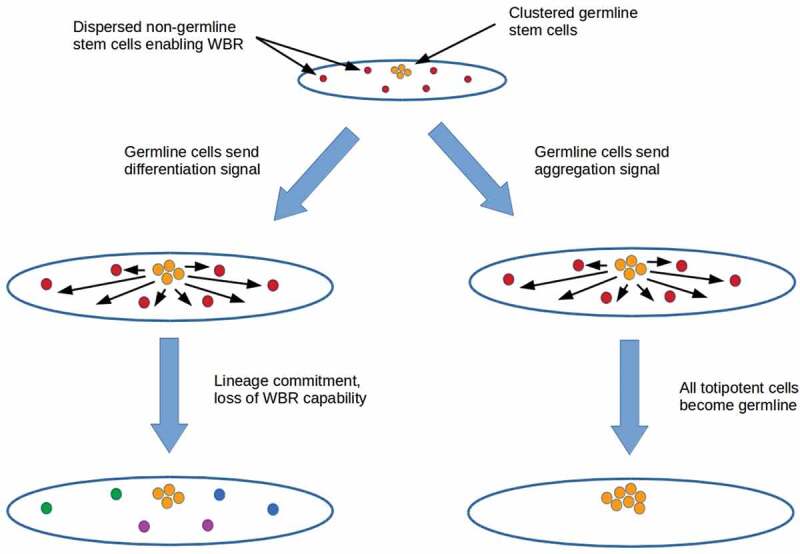
Two mechanisms for suppressing totipotency outside the germline in an ancestral lineage with both sequestered germline stem cells and dispersed non-germline stem cells enabling WBR. Forcing lineage commitment (left branch) suppresses totipotency outside the germline and hence WBR while leaving enabled at least partial somatic cell replacement by lineage-committed stem cells. Co-opting all totipotent cells to the germline (right branch) or killing non-germline stem cells suppresses not only WBR but also somatic cell replacement

The possibility that germline cells engage in coercive or deceptive signaling suggests two mechanisms for suppressing totipotency outside the germline: forced lineage commitment and co-option (Figure 4). Both depend on the prior sequestration of germline stem cells in an ancestral lineage that also had dispersed, non-germline stem cells enabling WBR. Signaling by germ cells to non-germline stem cells could be implemented by gonadally-produced hormones as discussed above, neurons, or both. Co-opting all totipotent cells into the germline via a migration-inducing signal (right branch in Figure 4) or alternatively, killing non-germline stem cells via induced apoptosis disables not only WBR but all somatic-cell replacement. Such a mechanism may have been active in the nematode lineage of organisms like *C. elegans*, which have (mostly) invariant somatic-cell lineages without cell replacement in adults [97].

An alternative to co-opting or killing non-germline stem cells is to force their commitment to a particular lineage (left branch in Figure 4). Forced lineage commitment disables WBR, but leaves open the possibility of somatic-cell replacement in at least some lineages. The presence of a wide variety of lineage-committed adult stem cells in mammals [e.g. humans, [98]] is consistent with this mechanism. Mice have been successfully cloned after injecting pluripotent embryonic stem cell nuclei into enucleated oocytes; embryonic stem cells alone are insufficient [99]. The inability of even pluripotent embryonic stem cells to support WBR under known circumstances in mammals suggests that totipotency has been suppressed cytoplasmically.

Winner-take-all mechanisms may also render facultative parthenogenesis unstable. Burke and Bonduriansky [23,24], for example, show that sexual conflict due to asymmetric mating strategies for males and females can drive facultatively parthenogenic populations either toward asexuality or sexuality. Sexual selection may also contribute to suppressing parthenogenesis; it is common even among hermaphrodites, where it appears to influence both genital morphology and mating strategies [100]. Concentration-dependent molecular switches regulating entry into meiosis or interactions between haploid cells may have similar effects at the cellular scale.

## Loss of WBR increases cancer susceptibility

Unlike sequestered, transcriptionally-quiescent [68] germline stem cells, non-germline stem cells must continuously monitor their environments to determine what if any somatic cells need replacement. Coercive or deceptive signaling by the germline or its gonadal intermediaries can be expected to dysregulate non-germline stem cells not only by suppressing totipotency but also by blocking or altering the interpretation of signals that would otherwise induce appropriate regenerative responses.

The PIWI/piRNA system functions both to preserve genome integrity by suppressing transposon activity and to regulate gene expression both transcriptionally and post-transcriptionally [29,30]. This system is active in non-germline stem cells supporting WBR in characterized WBR-capable organisms [9]; hence it is reasonable to assume that it was active in the non-germline stem cells of WBR-capable ancestors of currently obligate-gametic, WBR-incapable lineages such as mammals. As an apparent enabler of WBR, the PIWI/piRNA system is a plausible target of coercive or deceptive signaling by the germline during winner-take-all competition.

While the expression and functions of the PIWI/piRNA system in somatic cells, including adult stem cells, of obligate-gametic, WBR-incapable animals remain poorly characterized, dysregulation of this system, particularly aberrant expression of piRNAs, is known to be associated with multiple mammalian cancers [47]. De-repression of transposons is one possible mechanism for cancer induction [101]; altered sensitivity to hormones, growth factors, or other signals is another. We suggest that a third possible mechanism is alteration of the internal cellular representation of expectations regarding microenvironment structure or state (implemented by gene-regulatory, metabolic, bioelectric, or cytoskeletal networks that process information). Such a representation is required by models of cells as Bayesian agents [55,58], but is as yet uncharacterized in stem cells. The finding that perturbations of cellular circadian rhythms, which are critical reference frames for cellular measurements of duration, increase cancer susceptibility [102] supports this suggestion.

Robust WBR, or even organ- or tissue-specific regeneration, is in some cases able to clear cancer cells or reprogram them to normalcy [48,49]. Hence non-germline stem cell dysregulation is not only capable of inducing cancers, but also removes a line of defense of the body against cancers.

## Conclusions

While the divergence of sexual from asexual lineages and the extinction of asexual ancestors in the lineages leading, e.g. to birds and mammals would be expected to occur on evolutionary timescales, competition between germline and non-germline stem cell lineages is postulated to occur ontogenetically and hence to be observable in the laboratory. It is expected to be most evident in taxa that are anatomically and physiologically similar to ancestral taxa at branchpoints between facultative sexuals with WBR and obligate sexuals without WBR. Obligate gametic reproducers with substantial, but no longer complete regenerative capacity, of which amphibians may be the most tractable examples, may also display direct evidence of germline versus non-germline stem cell competition.

Besides the competitive mechanism(s) employed, two principal questions are raised here: 1) under what environmental, developmental, or genomic conditions does winner-take-all competition between stem lineages arise? and 2) are there any conditions under which vegetative reproduction with facultative sex is evolutionarily stable? These questions are obviously closely related. The second asks whether lineages that appear to support stable combinations of WBR with facultative sex in fact do so; cnidarians may be the best lineage in which to address this question. The first asks how such stability is broken, and whether it can be broken experimentally in lineages that appear stable.

As noted earlier, the capability of WBR will be lost in a lineage if any change occurs that renders dispersed totipotent or pluripotent cells no longer capable of determining, from the information available to them in their microenvironments, what somatic cells need to be produced or replaced. If this happens during embryogenesis or post-embryonic development, aberrant juvenile or adult morphologies will result; these may or may not be viable or fertile. Many such changes in the local availability of information about target morphology are routinely introduced in the laboratory: homeotic mutants are an obvious example, but exogenous treatments that alter, e.g. bioeletcric fields can have similar effects [103,104]. Such experiments suggest that any transition from a dispersed to a centralized encoding of target morphology can disrupt the capability for WBR by removing local sources of information, even just in adulthood. The obvious candidate for a centralized encoding of target morphology is the central nervous system (CNS), which in any behaving animal must maintain some representation of the body’s structure and capabilities. Both evolutionary and developmental analyses, and well as studies in cancer, suggest that the CNS encodes morphological information and contributes to the active, long-distance control of morphological development by controlling cell proliferation and differentiation [105]. One might expect, therefore, that increasing elaboration of the CNS in any lineage will correlate with both decreasing regenerative capability and a transition to obligate sex. Such a correlation is indeed observed in bilaterian lineages.

These considerations together suggest a number of experimental approaches.

- Does the PIWI/piRNA system function to suppress transposon activity in non-bilaterian lineages and in basal bilaterians? If not, what are its ancestral functions? Can the onset of obligate sexuality be linked to an increase in transposon loading of or a difference in transposon families, e.g. DNA versus RNA, present in genomes, at least in some lineages?
- Steroid hormone receptor genes are ancestral in bilaterians, but both genes and functions have been lost in some invertebrate lineages [106]. When were steroid hormones co-opted as sex hormones? Kudikina *et al*. [107] report effects of exogenous steroids on regeneration in *Girardia*, a flatworm. Are such effects observed in other animals exhibiting WBR? What is the mechanism of action?
- Can regenerative capacity be enhanced by inhibiting sexualization, e.g. in regeneration-deficient planaria? If migration of germline progenitors to the nascent gonads is disrupted in *Drosophila* or a variety of vertebrates, the misdirected progenitors de-differentiate or undergo apoptosis [108,109]. Is regenerative ability improved in these systems?
- Can WBR be disrupted by disrupting nervous-system organization or function in WBR-capable animals such as planaria? Can cancer susceptibility be increased by disrupting CNS function? If so, are the disruptions consistent with CNS encoding of target morphology?
- Non-germline stem cells can only compete with germline stem cells if they are present in the body. All animal known to exhibit WBR have resident totipotent stem cell populations. In animals capable of only partial regeneration, however, stem cells may be produced transiently, at the wound site, by dedifferentiation of previously-committed cells [e.g. muscle cells in newts, [110,111]. Do any animals capable of WBR employ a dedifferentiation mechanism to generate the needed stem cells, or is dedifferentiation a later-evolving mechanism?
- Some organisms, e.g. *C. elegans* as noted earlier, lack non-germline stem cells as adults. What is the maximal level of anatomical and morphological complexity that can be maintained without adult stem cells? Do all organisms capable of producing stem cells by dedifferentiation also have standing populations of non-germline stem cells, or is transient, ad hoc stem-cell production sufficient to maintain an anatomically-complex organism?

In summary, we suggest that internal competition between stem-cell lineages destabilizes the ancestral animal – indeed, microbial – strategy of vegetative reproduction and facultative sex. Reproductive arms races with external threats follow from, and are dependent on, this internal arms race. Developmental mechanisms lock in the winning strategy, particularly in obligate sexual systems. Obligate sexuality eliminates WBR capability almost by definition, with increased susceptibility to and decreased ability to combat cancers as an inevitable side-effect.
